# The New Nurse

**Published:** 1920-03-20

**Authors:** 


### THE MATRONS' AND SISTERS' DEPARTMENT.

### THE NEW NURSE.

The present amicable controversy between the Chairman of the Lambeth Infirmary and the Health Ministry is of great interest to every member of the nursing profession. It has arisen, as our readers know, on account of the desire of the infirmary authorities to introduce an eight-hour day for their nurses, simultaneously with the discovery that they have no quarters in which to house the additional nurses required. The same condition of affairs exists in a large number of Poor-law institutions and voluntary hospitals. Everywhere there is a call for more probationers than the Nurses' Home can accommodate. Everywhere there is a difficulty in either building or renting a suitable house for extra probationers. In this dilemma Mr. Frank Briant, M.P., is making a strenuous effort to introduce the living-out system for some at least of the probationers, but the Health Ministry has refused so far to sanction the proposal.

Mr. Briant believes that the time has come for the evolution of a new type of nurse. He not only believes in the living-out system as an emergency expedient, he has persuaded himself that it is a better thing for the nurse and for the patients. He would like to see it become the rule. The proposals his Board have agreed to are as follows. The required probationers are to be under contract, like medical students, to present themselves at such hours and for such periods as may be laid down for them, an eight-hour day being observed, and presumably attendance at lectures and classes being required over and above the specified hours. Beyond these obligations the probationers' time is at their own disposal. They sleep at home. Such meals as fall within their eight hours spell of work would necessarily, we imagine, be taken at the institution. For others they would make their own arrangements.

The salary Mr. Briant offers to the living-out probationers is £2 2s. a week for the first year, £2 4s. for the second year, £2 7s. 6d. for the third year. Probably Mr. Briant has taken steps to ascertain that the women he wants are willing to enter for the infirmary course on these terms. When the enormous cost of building and equipping nurses' homes in the present day is taken into consideration, there is no doubt that the institution will pay no more on these terms than it would if it provided sleeping and living accommodation for its pupils.

We await with interest the working out of this knotty problem. The more it is discussed the better. The desirability of allowing probationers to live at home during their training has often been advocated, and during the war it seemed to many that a successful trial had been made of the system, such as demonstrated once for all its advantages. But those who advocate its introduction into hospitals forget that wherever large numbers of V.A.D. members were employed it became at once necessary to establish a hostel for them. Comparatively few institutions for the sick are situated in the midst of a residential neighbourhood, inhabited by likely probationers. It may be safely predicted that the Lambeth Infirmary itself would at once have to deal with applications from candidates residing at a distance, and what arangements would it make for them? The trials of cheap lodgings would wear down the strength of the strongest probationer returning, as she must do, at different times of the night and day in need of an immediate meal and a hot bath.

But the hospital nurses' home is not merely a convenient hostel. It is a protection and support to pupils in learning the secret of a new life. It is not possible to take hold of nursing with the left hand, to go on living at home, the useful sister and daughter, loaded with duties and cares dissociated from the chosen calling, the prey of all those who are on the lookout for "some one to lean on." It is a calling which demands all that the student has to give during these pupil years. Women whose minds are bent on social pleasures need not think that with an eight-hour day they can run on in the old way, filling up their off-time with continual engagements, and yet be fresh and vigorous for their daily duty. Many a medical student runs to seed in the difficult endeavour to lead two lives, and most are glad enough to have residential quarters in their school when available. We know a V.A.D. member who for two years lived at home in Wimbledon and in alternate months put in day and night work in Kensington; the war ended just in time for her. It is only in special quarters away from home that the probationer is set free from that multiplicity of interests which Mr. Briant desires for his probationers. In practice such interests are discovered uselessly to exhaust the vitality. No! If any must sleep out why not the sisters?

